# A Compact Impact Rotary Motor Based on a Piezoelectric Tube Actuator with Helical Interdigitated Electrodes

**DOI:** 10.3390/s18072195

**Published:** 2018-07-07

**Authors:** Liling Han, Huining Zhao, Haojie Xia, Chengliang Pan, Yizhou Jiang, Weishi Li, Liandong Yu

**Affiliations:** School of Instrument Science and Opto-electronics Engineering, Hefei University of Technology, Hefei 230009, China; han-liling9117@mail.hfut.edu.cn (L.H.); hnzhao@mail.hfut.edu.cn (H.Z.); hjxia@hfut.edu.cn (H.X.); clpan@hfut.edu.cn (C.P.); jiangyizhou1@mail.hfut.edu.cn (Y.J.); weishili@hfut.edu.cn (W.L.)

**Keywords:** piezoelectric, rotary, impact drive, LuGre model

## Abstract

This paper presents a novel impact rotary motor based on a piezoelectric tube actuator with helical interdigitated electrodes which has a compact structure and high resolution. The assembled prototype motor has a maximum diameter of 15 mm and a length of 65 mm and works under a saw-shaped driving voltage. The LuGre friction model is adopted to analyze the rotary motion process of the motor in the dynamic simulations. From the experimental tests, the first torsional resonant frequency of the piezoelectric tube is 59.289 kHz with a free boundary condition. A series of experiments about the stepping characteristics of different driving voltages, duty cycles, and working frequencies are carried out by a laser Doppler vibrometer based on a fabricated prototype motor. The experimental results show that the prototype rotary motor can produce a maximum torsional angle of about 0.03° using a driving voltage of 480 V_p-p_ (peak-to-peak driving voltage) with a duty ratio of 0% under a small friction force of about 0.1 N. The motor can produce a maximum average angle of about 2.55 rad/s and a stall torque of 0.4 mN∙m at 8 kHz using a driving voltage of 640 V_p-p_ with a duty ratio of 0% under a large friction force of about 3.6 N. The prototype can be driven in forward and backward motion and is working in stick-slip mode at low frequencies and slip-slip mode at high frequencies.

## 1. Introduction

A piezoelectric motor has the advantages of a simple structure, convenient control, easy miniaturization, a high displacement resolution, a fast response speed, and miniature precision actuation, which has been widely studied by many researchers [1,2]. According to the working principle, piezoelectric motors can be roughly divided into four types [3,4,5,6,7,8], including standing wave, travelling wave, inchworm, and impact motors. The standing wave and travelling wave motors are collectively called an ultrasonic motor. The ultrasonic motors work under the conditions of resonance and most of them have a high speed, high output force, and miniaturized structure, but their resolution is usually low. The inchworm motors have a large output force and steady step, but their structures are usually complex and their speed of motion is usually low. The impact piezoelectric motors transfer the micro vibration of the piezoelectric component to the single-directionally continuous motion of the output shaft by friction transmission and overcome the shortcoming of the small stroke of the piezoelectric component itself.

The piezoelectric motor can achieve linear motion, rotary motion, and linear-rotary motion according to their output motions [9,10,11,12,13,14]. There are many ways to realize the torsional displacement with piezoelectric motors nowadays. The torsional deformation of a piezoelectric motor can be achieved from the shear strain (piezoelectric coefficient *d*_15_) effect. The motor is often driven by the divided electrodes of piezoceramic and the production process is complex [15]. A micro actuator using plane bending deformation can also achieve structural torsion, and the structure is simple, but the output displacement is low [16]. Now, with the development of micro and nano scale processing technology, many researchers find that a super-helix and grooved metal structure can couple torsional vibration components [17,18,19]. Lee proposed a structure of an anisotropic composite plate [20] which coupled with the torsional deformation component based on the principle of strain transformation. Fuda firstly proposed a torsional actuator with a helical interdigitated electrodes (HIDEs) structure [21]. Following this, the torsional actuators driven by interdigitated electrodes (IDEs) have been widely studied [22,23]. Pan proposed a piezoelectric fiber actuator with a small size, with a diameter of 1 mm, and with a pair of HIDEs twisted on the outside surface of the piezoelectric fiber tube [24,25]. Pan also proposed novel discal piezoelectric transducers with spiral IDEs on the surfaces of the piezoelectric disks [26]. Zhang proposed a piezoelectric rotary motor with two pairs of grooved HIDEs of a piezoelectric solid cylinder [27].

This paper is a further study of the HIDEs of piezoelectric motors. A novel compact impact rotary motor based on a piezoelectric tube actuator with HIDEs which increase the output capability is developed. The prototype motor with the core of HIDEs on the outside surface with the angle of 45 degree of the piezoelectric tube actuator has been designed, fabricated, tested, and compared with the simulations. In Section 2, the basic working principle of the rotary motor, structure design and parameters, and fabrication are described. Then, the simplified dynamic model is analyzed, and the friction model of the LuGre model is introduced into the analysis in Section 3. In Section 4, the finite element method (FEM) analysis of the piezoelectric tube motor is researched at first, the impedance is then measured, and the stepping characteristics and loading capacity of the prototype motor are tested and contrasted to the simulation results in this section. In Section 5, the discussions and conclusions are introduced in detail.

## 2. Working Principle, Structure Design, and Fabrication

**Figure 1:**
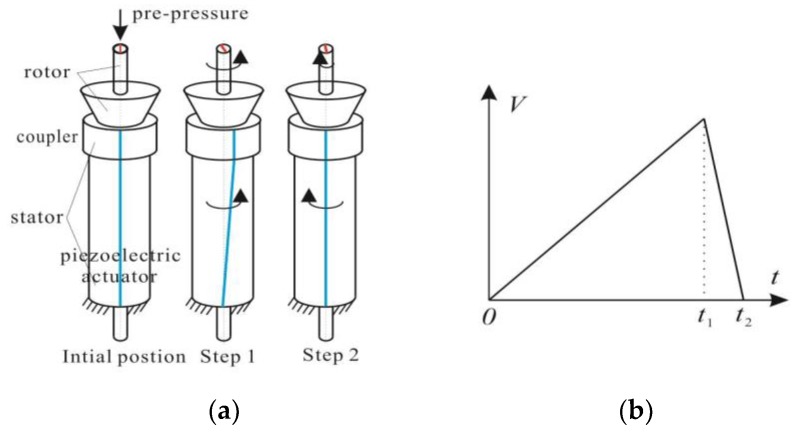
Working process of an impact rotary motor: (**a**) Working principle; (**b**) Driving signal.

The rotary motor usually consists of a stator and rotor. The driving signal and simplified working principle of the motor are shown in Figure 1. Figure 1b shows the saw-shaped driving voltage. The pre-pressure is applied to the contact face between the stator and rotor to provide friction force and supporting force. There are two different steps in one working cycle. In the first step (0–*t*_1_): the driving voltage increases slowly, the piezoelectric actuator produces torsional displacement as the coupler is adhered to the piezoelectric actuator, and then the coupler produces the same counterclockwise rotation with the piezoelectric actuator. The maximum static friction force is large enough to ensure the synchronized counterclockwise motion of the shaft and actuator. In the second step (*t*_1_–*t*_2_): the driving voltage falls off suddenly, and the actuator rapidly moves clockwise to its original position. However, the shaft will produce a decelerated clockwise motion. The reverse accelerations of the shaft and actuator are different. In short, the shaft rotates one big counterclockwise angle and pulls back the shaft by a small clockwise angle. After one working cycle, the shaft will produce a counterclockwise motion. When repeating the working cycle quickly, the shaft will continuously move counterclockwise. When the time of *t*_1_ is much shorter than *t*_2_, the shaft can produce a clockwise motion. The driving frequency is often very low under this motion, thus the impact motor can also be called the stick-slip motor under this condition. However, the working principle will be different at a higher driving frequency, the stiction in step 1 will not exist, and the shaft is accelerated by sliding friction. The motor will then be operated in slip–slip mode [28,29].

**Figure 2:**
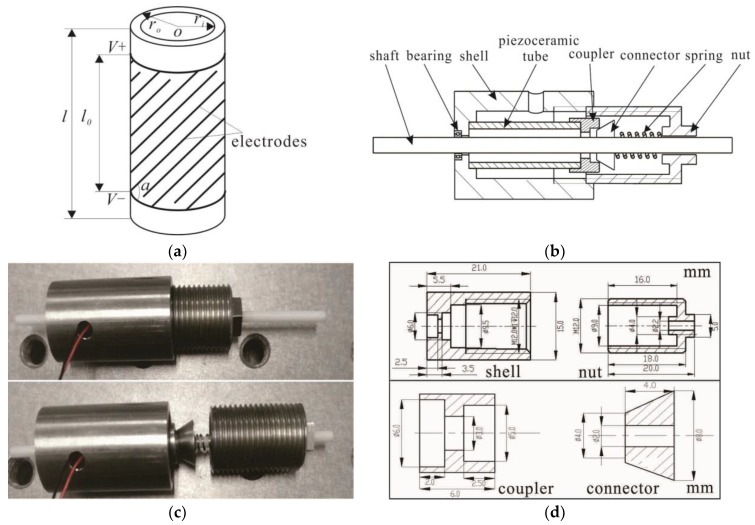
Structure of the rotary motor: (**a**) Schematic diagram of the piezoelectric tube; (**b**) Cross-sectional view of the motor model; (**c**) The prototype motor; (**d**) Dimensions of the shell, nut, coupler, and connector.

Figure 2a shows the piezoelectric tube actuator which is the key part of the motor, and Figure 2b shows the structure of the impact rotary motor. The motor consists of a piezoelectric tube, a shell, a bearing, a coupler, a connector, a spring, a nut, and a shaft.

Figure 2c shows the fabricated prototype motor which has a maximum diameter of 15 mm with a length of 65 mm. The piezoelectric tube actuator is of the lead Zirconate Titanate (PZT) type (YT-5L) of dimensions *l* = 15 mm, *r_o_* = 3 mm, and *r_i_* = 2.5 mm. The piezoelectric tube actuator has five pairs of HIDEs with the effective length (*l*_0_) of 9 mm and the helical angle of the electrodes (*α*) is 45°. The electrodes of the piezoelectric tube are isolated from UV curable green oil. The connector and shaft are adhered by epoxy resin mixed with glass powder, and other parts that need to be adhered using the same way. The shaft is made of zirconia ceramic with the diameter of 2 mm and length of 65 mm. The coupler is glued to the piezoelectric tube to protect the piezoelectric tube. The connector is a conical tube which is glued to the shaft in order to transfer the motion of the piezoelectric tube through line contact. The piezoelectric tube and coupler work as the stator. The connector and shaft work as the rotor. The bearing plays a supporting role. By changing the axial position between the nut and shell through a spring, the pre-pressure between the stator and rotor can be adjusted. The sectional view and related dimensions of the shell, nut, coupler, and connector are illustrated in Figure 2d.

According to the recent reference, the rotational angle of the piezoelectric tube under quasi-static driving can be expressed as Equation (1) [30]:

(1) $$\theta =\frac{2k(d_{31}-d_{33})l_{0}nV\cos\alpha }{\pi r_{o}{}^{2}}$$

where *d*_33_ and *d*_31_ coefficients produce piezoelectric strains; *l*_0_ is the length of effective electrodes; *r_o_* represents the outer radii of the tube; *V* represents the driving voltages between the adjacent HIDEs; *α* is the helical angle of the electrodes; and *k* is the compensation coefficient, often between 0.5 to 1, which is produced by the non-uniform distribution of electric field between the adjacent electrodes and the non-uniform radial strain in the of the piezoelectric tube. By adjusting the pairs of electrodes (*n*), the driving abilities of rotational angles will be optimized.

Table 1 shows the referenced material parameters of the rotary motor.

## 3. System Modeling and Dynamic Simulation

**Figure 3:**
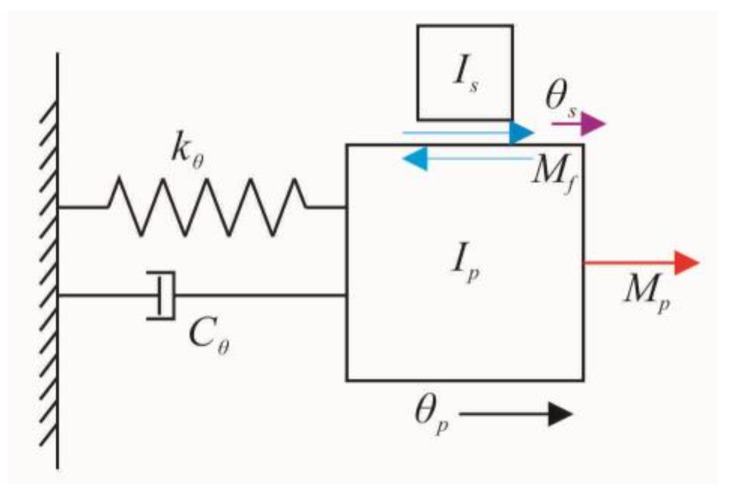
Simplified model of the prototype motor.

Through the diagram of the impact rotary motor, the prototype motor can be analyzed using a spring-mass-damper system, as shown in Figure 3. The piezoelectric tube actuator and coupler are simplified as a spring-mass-damper and the connector and shaft are simplified as mass.

The kinetic equations can describe the working process of the motor [31]:

(2) $$\left\{ \begin{array}{l} I_{p}{\theta^{\prime \prime }}_{p}=M_{p}-C_{\theta }{\theta^{\prime }}_{p}-k_{\theta }\theta_{p}-M_{f}\\ I_{s}{\theta^{\prime \prime }}_{s}=M_{f}\\ M_{p}=k_{\theta }\theta_{p}=\delta V \end{array}\right.,$$

where *C_θ_*, *k_θ_*, *I_p_*, and *θ_p_* are the torsional damper coefficient, elastic stiffness, equivalent moment of inertia, and the rotational angle of the stator (including piezoelectric tube and coupler), respectively; *I_s_* and *θ_s_* are the moment of the inertia and rotational angle of the rotor (including shaft and connector), respectively; *M_p_* is the driving torque; *M_f_* is the friction torque; and *δ* is the conversion coefficient between *M_p_* and applied voltage *V*.

Supposing that the torsional elastic stiffness of the stator is equal to the torsional elastic stiffness of the piezoelectric tube actuator because the torsional stiffness is mainly provided by the piezoelectric tube actuator, the torsional elastic stiffness can be deduced from the following equation using the basic torsional resonant frequencies of the piezoelectric tube actuator:

(3) $$f_{r}=\frac{1}{2\pi }\sqrt{\frac{k_{\theta }}{I_{p}}},$$

where *f_r_* is the first mode resonant frequency of the piezoelectric tube, and *I_p_* is the equivalent moment of inertia of the piezoelectric tube.

The equivalent moment of inertia of the stator can be obtained by the equation:

(4) $$I_{p}=\frac{1}{3}I=\frac{1}{3}\cdot \frac{1}{2}m\left(r_{o}{}^{2}+r_{i}{}^{2}\right)=\frac{1}{6}\rho \pi \left(r_{o}{}^{4}-r_{i}{}^{4}\right),$$

where *I* is the moment of inertia of the piezoelectric tube using the equation of the moment of inertia of the cylinder, *m* is the mass of the piezoelectric tube, and *ρ* is the density of the piezoelectric tube.

Then torsional damper of the stator can be obtained by the equation:

(5) $$\xi =\frac{C_{\theta }}{2\sqrt{k_{\theta }I_{p}}}=\frac{1}{2Q},$$

where *ξ* is the damping ratio and *Q* is the quality factor, which is calculated by the impedance curve.

The LuGre model is a common dynamic friction model which describes phenomena of Coulomb friction, pre-sliding, variable static friction, Stribeck friction, and friction lag with first order differential equations [32]. The LuGre model has been widely used in contact friction modelling. The rotational LuGre friction model can be described by:

(6) $$\left\{ \begin{array}{l} M_{f}=\sigma_{0}z+\sigma_{1}\frac{dz}{dt}+B\theta^{\prime }\\ \frac{dz}{dt}=\theta^{\prime }-\frac{\left|\theta^{\prime }\right|}{g(\theta^{\prime })}z\\ g(\theta^{\prime })=\frac{T_{c}+(T_{s}-T_{c})e^{-{\left(\theta^{\prime }/{\theta^{\prime }}_{s}\right)}^{\alpha }}}{\sigma_{0}} \end{array}\right.,$$

where *θ*′ is the relative rotational angular velocity between the two surfaces in contact; *θ*′*_s_* is the Stribeck angular velocity; *z* is the average bristle deflection; *σ*_0_ is the stiffness; *σ*_1_ is a damping coefficient; *B* is the viscous friction parameter; *g*(*θ*′) is the angular velocity dependent function with a Stricbeck effect; *T_s_* is the maximum static friction torque; *T_c_* is the Coulomb friction torque; and *M_f_* is the predicted friction torque. The value of *α* = 2 is suggested in [33].

**Figure 4:**
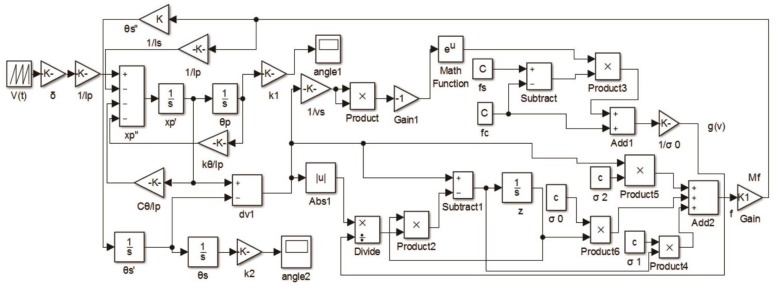
Dynamic simulation block of the rotary motor.

According to the Equations (2) and (6), the dynamic simulation model of the proposed impact rotary motor is established, and the simulation process can be obtained from Figure 4 [34].

## 4. Prototype Tests and Results

### 4.1. Analysis of Piezoelectric Tube Actuator and Simulation Parameters

**Figure 5:**
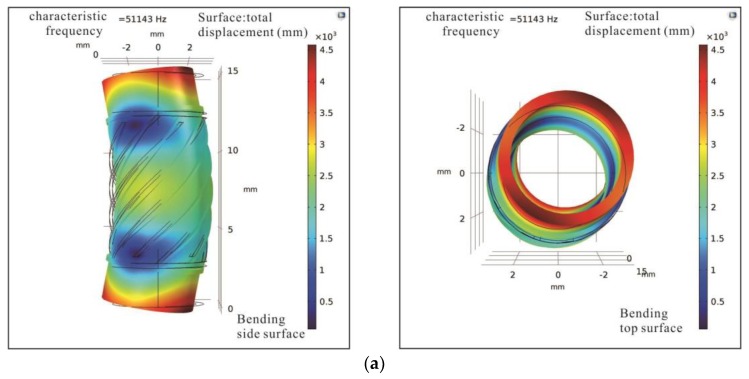

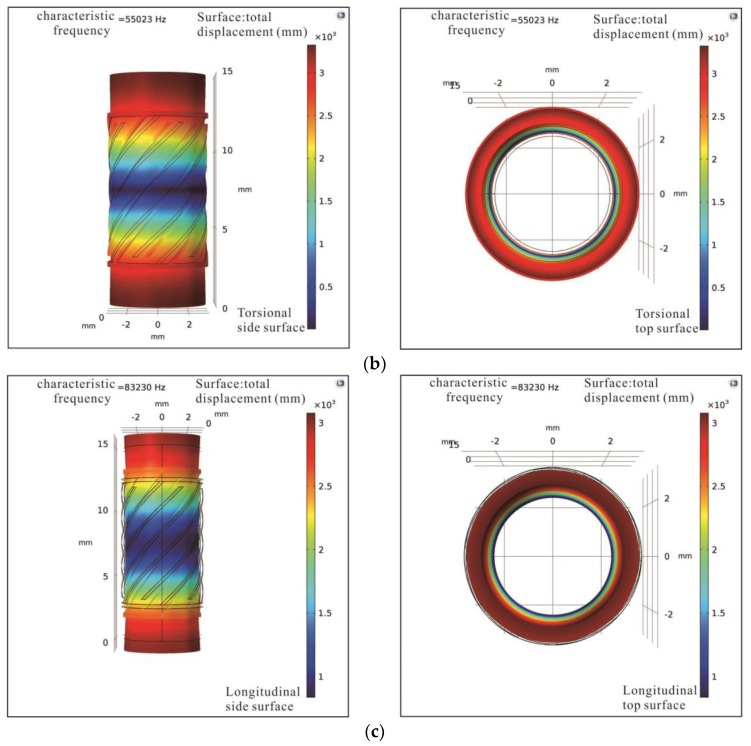
FEM analysis results of the piezoelectric tube actuator showing the side and top view: (**a**) First bending vibration mode of 51.143 kHz; (**b**) First torsional vibration mode of 55.023 kHz; (**c**) First longitudinal vibration mode of 83.230 kHz.

A finite element method (FEM) analysis of the piezoelectric tube actuator is conducted in Comsol Multiphysics, where the geometry of the piezoelectric tube is built in SolidWorks software and imported to Comsol Multiphysics. There is a free boundary condition applied in the simulation analysis to extract basic modes of bending, torsional, and longitudinal vibrations, as shown in Figure 5. The first bending, torsional, and longitudinal vibration modes are 51.143 kHz, 55.023 kHz, and 83.230 kHz, respectively.

**Figure 6:**
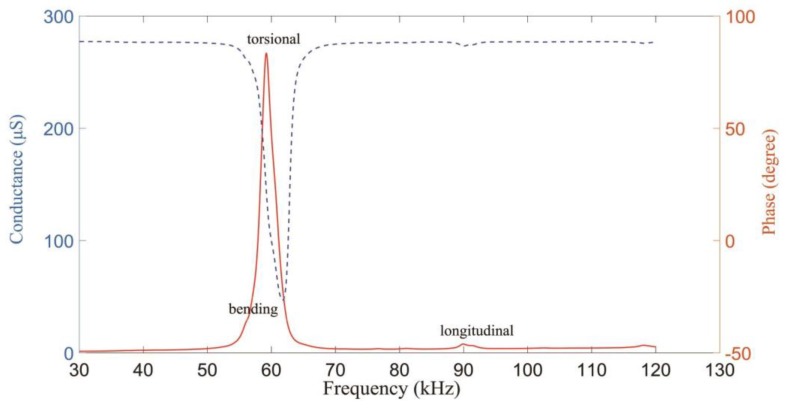
Dynamic abilities of the piezoelectric tube actuator.

The LCR-8105G analyzer (GWINSTEK) is used to test the dynamic abilities. The piezoelectric tube actuator is free during measuring. The analyzed performance is shown in Figure 6. According to the modal analysis from the simulation, it can be seen that the first bending, torsional, and longitudinal vibration modes occur at 56.586 kHz, 59.289 kHz, and 89.502 kHz, respectively. Compared with the measured three modes, the peak value of the torsional mode is the biggest. That means the torsional deformation is the major motion under the quasi-static driving signal.

The first mode resonant frequency (*f_r_*) is 59.289 kHz and the quality factor (*Q*) is 40 according to the test result of the piezoelectric tube in Figure 6, and the simulation parameters of Figure 4 are shown in Table 2 according to Equations (3)–(5).

### 4.2. Stepping Characteristics of the Motor

**Figure 7:**
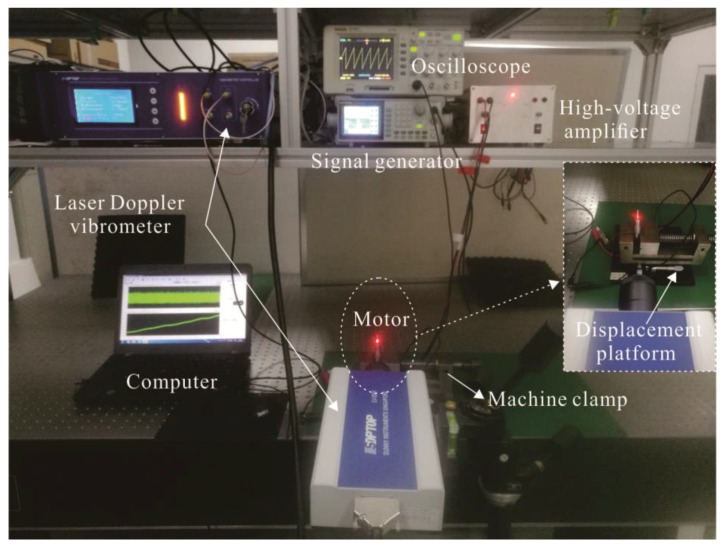
The testing system of the rotary motor.

The velocities and displacements of the prototype motor with no load are tested by a laser Doppler vibrometer. Figure 7 shows the testing system. An original saw-shaped driving signal is produced by using a signal generator (AFG-2225, GWINSTEK). Then, the saw-shaped signal amplified through a high-voltage amplifier is used to drive the motor. The high-voltage amplifier takes the PA 94 as the core. The oscilloscope is used to observe the saw-shaped driving signal which is applied to the piezoelectric tube actuator.

**Figure 8:**
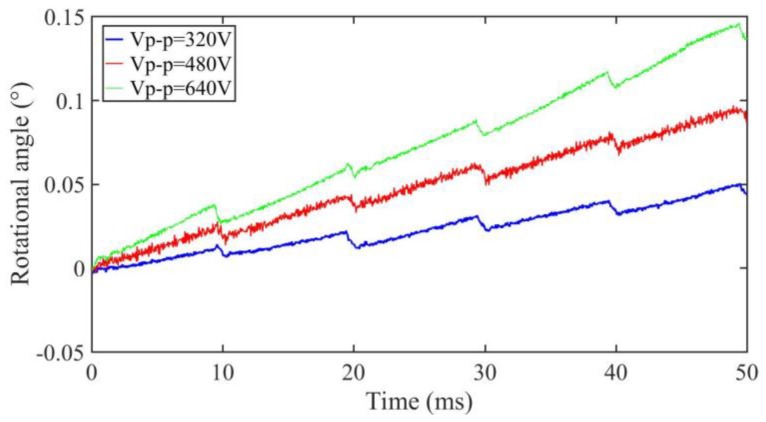
Stepping characteristics of the prototype motor under different driving voltages.

A small pre-pressure of about 0.3 N is provided by the spring and the small friction force is tested at about 0.1 N. Then, the maximum static friction torque (*T_s_*) and Coulomb friction torque (*T_c_*) in the simulation can be set to 2.6 × 10^−4^ N∙m and 2.2 × 10^−4^ N∙m, respectively. The outside surface of the shell is firmly fixed by a machine clamp during the entirety of the experimental tests. The forward output stepping characteristics of the motor when driving in different voltages (320 V_p-p_, 480 V_p-p_, and 640 V_p-p_) at 100 Hz with a duty ratio of 0% can be obtained in Figure 8. The experimental maximum torsional angle of the stator is nearly 0.03° under a driving voltage of 480 V_p-p_, and the compensation factor *k* is 0.75 when taking the measured angle into the Equations (1) and (2), and thus the simulation conversion coefficient (*δ*) can be calculated as −3.72 × 10^−4^ N∙m/V; thus, all the parameters in the simulation are calculated. The motor is working in stick-slip mode at 100 Hz. The average stepping rotational angles are about 0.008°, 0.017°, and 0.028° under a 320 V_p-p_, 480 V_p-p_, and 640 V_p-p_ driving voltage, respectively (see Figure 8). The results show that the stepping angle of the shaft will increase when the driving voltage increases and the stepping angle is approximately proportional to the driving voltage.

**Figure 9:**
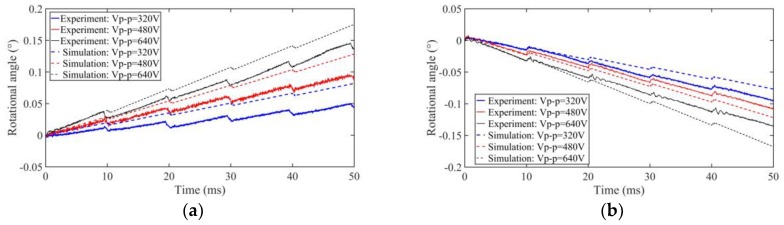
Stepping characteristics of the prototype motor under different driving voltages in experiments and simulations: (**a**) forward; (**b**) backward.

Taking the simulation parameters to the simulation block of Figure 4, the stepping characteristics of the motor can be analyzed. The simulation and experimental results of forward (duty ratio 0%) and backward (duty ratio 100%) motion can be shown in Figure 9 and be compared when driving in 320 V_p-p_, 480 V_p-p_, and 640 V_p-p_ voltages at 100 Hz. The simulation results have the same motion trend as the experimental results. Because of the imperfect processing technology of the piezoelectric tube, installation accuracy, manufacturing error, periodic thermal wear, and measurement error in experimental tests, there is difference between the simulation and experimental results.

**Figure 10:**
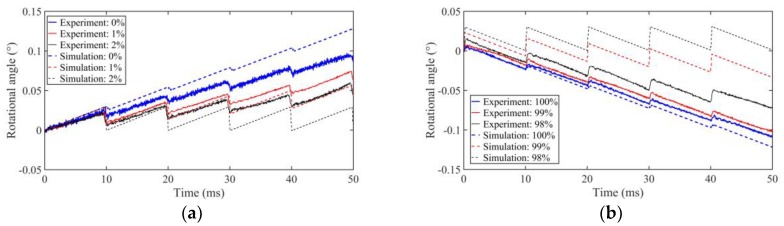
Stepping characteristics of the prototype motor under different duty cycles in experiments and simulations: (**a**) forward; (**b**) backward.

The driving voltage is fixed at 480 V_p-p_ with the driving frequency of 100 Hz, and the output stepping characteristics of the motor are shown in Figure 10 when the duty cycle of driving voltage is 0%, 1%, 2%, 98%, 99%, and 100%, respectively. When the duty cycle is 98% and 2%, the motor displays nearly no motion in the simulation, but it has a high output angle in experiment, which means that the motor can work in a wide duty cycle at a low frequency in practice. The average stepping rotational angles are about 0.017°, 0.009°, and 0.006° with a driving duty ratio of 0%, 1%, and 2%, respectively (see Figure 10a).

**Figure 11:**
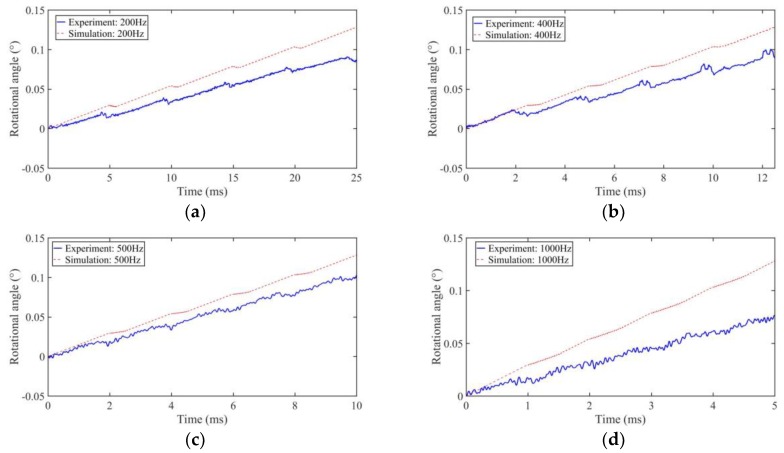
Stepping characteristics of the prototype motor under different driving frequencies in experiments and simulations: (**a**) 200 Hz; (**b**) 400 Hz; (**c**) 500 Hz; (**d**) 1 kHz.

When the driving voltage is fixed at 480 V_p-p_, the duty cycle is 0%, and the output stepping characteristics of motor are shown in Figure 11 when the driving frequency is 200 Hz, 400 Hz, 500 Hz, and 1 kHz, respectively.

In Figure 11, after five working cycles, the simulation results of the output angle are 0.128184°, 0.128185°, 0.12815°, and 0.12801°, and the experimental results are 0.086°, 0.091°, 0.115°, and 0.075° at the frequencies of 200 Hz, 400 Hz, 500 Hz, and 1 kHz, respectively. The simulation results show that with the increase of the driving frequency and the decrease of the pull back angle, the motor will work in stick-slip motion at a low frequency and work in slip-slip motion at a high frequency. The experimental results have the same trend compared to the simulations results. From the experimental results, the motor is working in stick-slip mode below 400 Hz, and working in slip-slip motion above 500 Hz. The experimental results also show that the motor will have a large stepping output when it works in slip-slip mode at a suitable frequency of about 500 Hz, and a low stepping output when the driving frequency is higher than 1 kHz. This is because the small fixed friction torque will not be large enough to provide the accelerated motion speed under this circumstance.

**Figure 12:**
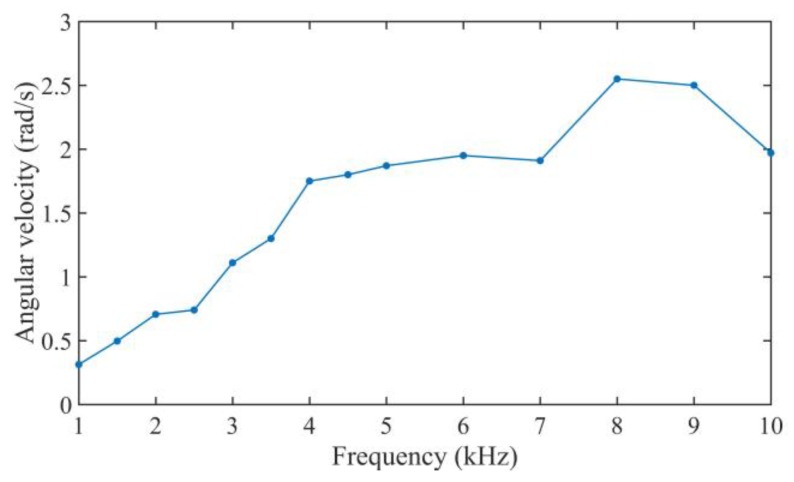
Average angular velocity of the motor.

When increasing the pre-pressure by tightening the nut, the friction force will be increased and it is tested at about 3.6 N. There is nearly no reverse motion in the rotary motion, and the curves of the rotational angle are nearly a smooth slant line. The average angular velocity of the motor is calculated at a frequency from 1 kHz to 10 kHz at a driving voltage of 640 V_p-p_ with a duty cycle of 0%, as shown in Figure 12. Increasing the driving frequency from 1 kHz to 8 kHz will increase the average angular velocity of the motor.

### 4.3. Loading Capacity

**Figure 13:**
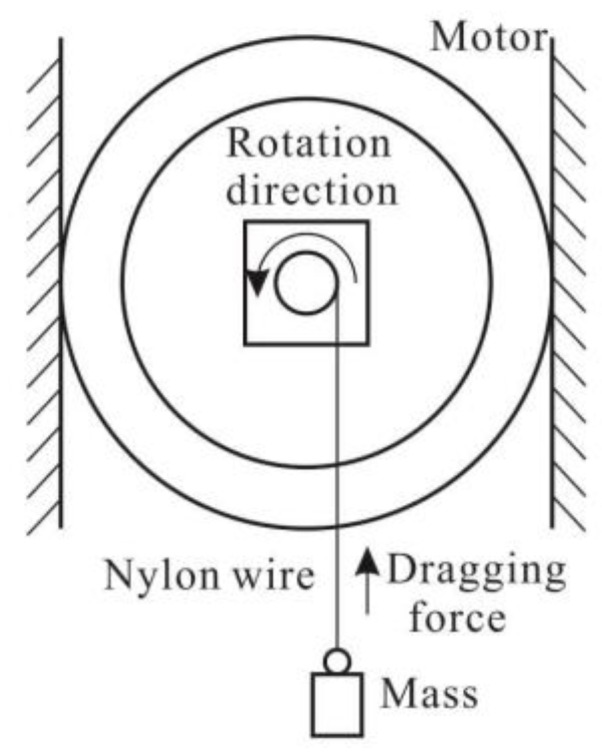
The testing system for load.

Loading capacity is an important indicator for evaluating the actuators. The applied load torque of the motor is generated through a mass which is connected to the shaft by a thin wire. The measurement system is shown in Figure 13.

**Figure 14:**
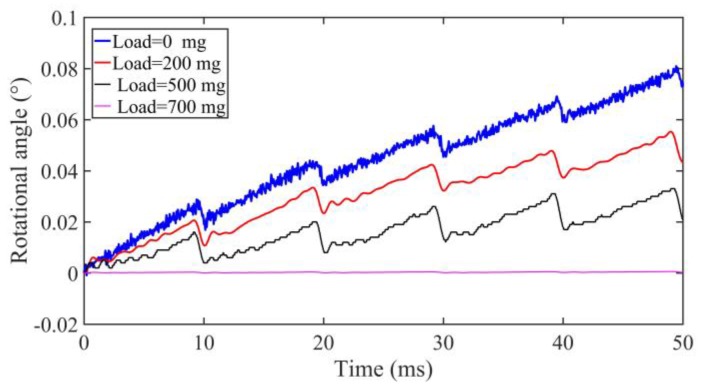
Rotational angle under different external loads at 100 Hz.

When the driving voltage is fixed at 480 V_p-p_ with the driving frequency of 100 Hz under the duty ratio of 0% at the small friction force of about 0.1 N, the output rotational angles of the motor are shown in Figure 14 under the external loads of 0 mg, 200 mg, 500 mg, and 700 mg, respectively. With the increase of load, the output angle of the rotating shaft will decrease. There is nearly no motion when the load is 700 mg.

**Figure 15:**
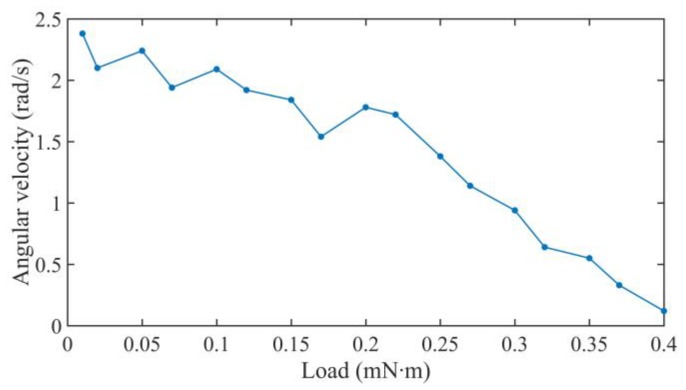
Rotational angle under different external loads at 8 kHz.

When the driving voltage is fixed at 640 V_p-p_ with the driving frequency of 8 kHz under the duty ratio of 0% at the large friction force of about 3.6 N, the average angular velocities of the motor are shown in Figure 15 under the different load torques. The maximum load torque of the motor is about 0.4 mN∙m, which is higher than the previous fiber torsional piezoelectric actuator [24,25]. The structure is simple compared to the impact motor using the piezoelectric coefficient d_15_ effect [15].

## 5. Discussions and Conclusions

This paper presents a novel compact impact rotary motor based on a piezoelectric tube actuator with helical interdigitated electrodes. The motor consists of a piezoelectric tube actuator, a shell, a bearing, a coupler, a connector, a spring, a nut, and a shaft. The pre-pressure between the stator and rotor is adjusted by changing the axial position between the nut and shell through the spring. According to the dynamic model of the motor, the rotational LuGre model is adopted for the simulations of the working behavior of the motor.

The stepping characteristics of different driving voltages, duty cycles, and working frequencies are carried out by a laser Doppler vibrometer. The experimental and simulation results are listed in the same figures. The two results show that the motor can obtain the largest stepping angle under a high driving voltage and high duty ratio at a fixed frequency. The LuGre friction model can describe the stick-slip motion at a low frequency and slip-slip motion at a high frequency, and the largest stepping angle can be obtained under slip-slip motion at an appropriate frequency. Because of the imperfect processing technology of the piezoelectric tube, installation accuracy, manufacturing error, periodic thermal wear, and measurement error in experimental tests, there is difference between the simulation and experimental results.

The prototype motor can work in forward and backward motion. The experimental results show that the prototype rotary motor can produce a maximum torsional angle of about 0.03° using a driving voltage of 480 V_p-p_ with a duty ratio of 0% and a small friction force of about 0.1 N, and produce a maximum average angle of about 2.55 rad/s, and the maximum load torque of the motor is about 0.4 mN∙m at 8 kHz with a large friction force of about 3.6 N. With the increase of the driving frequency and the decrease of the pull back angle, the motor works in stick-slip mode below 400 Hz and slip-slip motion above 500 Hz with low pre-pressure. In stick-slip mode, the minimum stepping rotational angle is about 0.006° under a driving voltage of 480 V_p-p_ with a driving duty ratio of 2% at 100 Hz.

The experimental results indicate that the compact prototype motor has high precision, a wide range of working frequencies, a good output speed, and good load torque. The proposed motor can be used in micro/nano positioning precision motion, micro operations, optical engineering, biomedical science, such as cell transport and the assembly of micro/nano components, and so on. The improved precise model to analyze a motor’s motion process and the improved structure will be studied in the future.

## Figures and Tables

**Table 1:** Material parameters of the motor.

Materials	Parameters	Value	Unit
PZT-5	Piezoelectric coefficient (*d*_31_)	−195	pC·N^−1^
	Piezoelectric coefficient (*d*_33_)	450	pC∙N^−^^1^
	Density (*ρ*)	7600	kg∙m^−^^3^
	Poisson’s ratio	0.29	
304 stainless steel	Density (*ρ*)	7930	kg∙m^−^^3^
Zirconia Ceramic	Density (*ρ*)	5850	kg∙m^−^^3^

**Table 2:** Parameters in simulation.

Parameters	Value	Unit
Torsional elastic stiffness *(k_θ_*)	342.7	N∙m
Torsional damper (*C_θ_*)	2.3 × 10^−5^	N∙m∙s
Equivalent moment of inertia (*I_p_*)	2.5 × 10^−9^	kg∙m^2^
Equivalent moment of inertia (*I_s_*)	5.3 × 10^−9^	kg∙m^2^
Maximum static friction torque (*T_s_*)	2.6 × 10^−4^	N∙m
Coulomb friction torque (*T_c_*)	2.2 × 10^−4^	N∙m
Stiffness (*σ*_0_)	260	N∙m/rad
Damping coefficient (*σ*_1_)	0.1 × 10^−1^	N∙m/(rad∙s^−1^)
Viscous friction parameter (*B*)	0	N∙m/(rad∙s^−1^)
Stribeck angular velocity (*θ*′*_s_*)	0.2	rad/s
